# The current status of psychological birth trauma in women who had a vaginal delivery and associated factors: a questionnaire-based, cross-sectional study

**DOI:** 10.3389/fpubh.2025.1539305

**Published:** 2025-02-17

**Authors:** Hong Qin, Weiwei Wei, Xiaoyan Feng, Xiaochang Yang

**Affiliations:** ^1^Department of Nursing, The First Affiliated Hospital of Chongqing Medical University, Chongqing, China; ^2^Department of Obstetrics, The First Affiliated Hospital of Chongqing Medical University, Chongqing, China

**Keywords:** puerperium, psychological birth trauma, postpartum post-traumatic stress disorder, vaginal delivery, influencing factors

## Abstract

**Objective:**

Individuals vary in their perception of psychological birth trauma (PBT), with some individuals progressing to postpartum post-traumatic stress disorder (PP-PTSD). However, from both preventive and developmental perspectives, PBT and PP-PTSD have received limited attention in China. This study examines the prevalence and influencing factors of PBT among women who underwent vaginal delivery in Chongqing, China, at 3 days and 42 days postpartum, as well as the correlation between perceived PP-PTSD and PBT, aiming to enhance understanding in this field.

**Methods:**

This questionnaire-based, cross-sectional study was conducted on women who had a vaginal delivery admitted to a grade III-A general hospital using convenience sampling between February and April of 2024. Participants were questioned using a general questionnaire, the Birth Trauma Perception Scale for Women During Vaginal Delivery (BTPS-WVD) scale, and the Postpartum Post-Traumatic Stress Disorder Scale (PP-PTSD) at 3 and 42 days postpartum. Univariate and multiple linear regression analysis was performed to identify factors associated with PBT at 42 days postpartum. Pearson correlation analysis was used to investigate the correlation between PBT and PP-PTSD in women who had a vaginal delivery.

**Results:**

The average score of PBT at 3 and 42 days postpartum were (43.37 ± 9.46) and (51.40 ± 13.54) respectively, the difference was statistically significant (*p* < 0.05). There were statistically significant differences in the dimensions of medical support trauma perception, delivery pain trauma perception, family support trauma perception, and delivery outcome trauma perception (*p* < 0.05). The average score of PP-PTSD at 3 and 42 days postpartum were (22.38 ± 7.13) and (22.29 ± 5.77) respectively, with no statistical significance (*p* > 0.05). The positive rate of PP-PTSD (score ≥ 38) at 3 and 42 days postpartum were 5 and 2%, respectively. Univariate analysis showed that, feeding mode, the effect of breast swelling on mood, mother separate from the newborn, separation time between mother and newborn, place of puerperium, psychological discomfort caused by delivering with others, use of epidural anesthesia, delivery time, advise others to deliver vaginally, the effects of wound pain, time of the postnatal wound pain and who decides on abnormal delivery were independently associated with PBT (*p* < 0.05). Multiple linear regression analysis showed that, mother separate from the newborn, separation time between mother and newborn, place of puerperium, psychological discomfort caused by delivering with others, the effects of wound pain, time of wound pain, who decides on abnormal delivery were independently associated with PBT (*p* < 0.05). Pearson correlation analysis showed that, PBT and its four dimensions were positively correlated with PP-PTSD (*r* = 0.488, *p* < 0.001).

**Conclusion:**

Women who experienced PBT during vaginal delivery reported significantly higher levels of perceived trauma at 42 days postpartum compared to 3 days postpartum. Clinical staff, family, and society should pay attention to the risk factors and take corresponding intervention measures to reduce the degree of PBT and promote maternal and child health.

## Introduction

Psychological Birth Trauma (PBT) refers to the painful emotional experiences that women go through during childbirth, such as intense fear, hopelessness, and a sense of losing control. This subjective experience originates in the labor process and its effects continue into the postpartum period (1). The prevalence of PBT varies from 20 to 68.6% worldwide (1, 2). PBT can lead to suicidal impulses (3), anxiety and sadness (4), and a decrease in the willingness to have another child due to fear (5). It can also increase the rate of non-medical indication cesarean sections in clinical practice, disrupt mother-baby, marital, and family relationships (6), and create postpartum mental health problems, substantially damaging the physical and mental development of the next generation (7). Approximately 3% of women develop Postpartum Post-Traumatic Stress Disorder (PP-PTSD) (8), exhibiting clinical symptoms such as traumatic memories, cognitive negativity, hypervigilance, or avoidance behaviors (9). Women with PP-PTSD are often also affected by postpartum depression (10). The Global Strategy for Women’s, Children’s, and Adolescents’ Health (2016–2030) (11) and the WHO recommendations on intrapartum care for a positive childbirth experience (12) both suggest that current and future maternal and child health services should prioritize optimizing the maternal experience throughout pregnancy and childbirth, as well as promoting maternal, fetal, and infant safety. Therefore, evaluating and monitoring PBT interventions is critical for maternal and child health, as well as family and societal development. Existing research has paid little attention to PBT itself, with only a few scholars reviewing the research on PBT (13–15).

In this study, the PBT of women who had vaginal deliveries was evaluated at 3 and 42 days postpartum, respectively, to understand the differences, explore whether the degree of PBT changes with postpartum time, and analyze its influencing factors, thereby providing a theoretical basis for effective clinical interventions.

## Methods

### Study design and participants

This questionnaire-based, cross-sectional study was conducted on women who had a vaginal delivery admitted to a grade III-A general hospital using convenience sampling between February and April of 2024. The team’s previous research examined the influencing factors of postpartum trauma within 3 days post-delivery. This current study extends that investigation to explore the influencing factors of postpartum trauma at 42 days post-delivery, comparing these factors with those observed within the initial 3 days to better understand the changes in postpartum trauma over time. The inclusion criteria were: (1) vaginal delivery; (2) single birth; (3) Gestational age ≥ 36 weeks; and (4) Neonatal Apgar score ≥ 7 points. The exclusion criteria were: (1) Postpartum hemorrhage occurs after delivery; (2) Perineal degree III and IV cleft; (3) Instrument-assisted delivery; (4) women with imperfect information; and (5) Who did not complete the scale assessment twice. The sample size was determined using the 10 EPV principle (16), considering multiple factors. A total of 30 independent variables were involved in this study. When EPV = 10, the required research objects were 300, and the incidence rate of PBT reported in literature was about 60%. The total required sample size was 300÷50% ≈ 180. Considering that there is a 20% shedding rate, in order to avoid errors as much as possible, the sample size is required to be 216 cases, and the final number of included samples is 306 cases. This study received approval from the Ethics Committee of the First Affiliated Hospital of Chongqing Medical University, China, and complied with ethical standards under ethics number K2024-082-01.

### Procedures

#### Basic characteristics questionnaire

The researchers designed a questionnaire specifically for this study, comprising the following sections: (1) demographic characteristics, such as age, work situation, education. (2) Birth-related information, including whether to use epidural anesthesia, newborn sex, the time of the postnatal wound pain, etc.

#### Birth trauma perception scale for women during vaginal delivery

This scale was developed by Lian Zerong (17) and is suitable for the cultural, reproductive, and medical backgrounds of China. The scale includes four dimensions: medical support trauma perception, delivery pain trauma perception, family support trauma perception, and delivery outcome trauma perception, with a total of 31 items. Respondents rated each item on a 5-point scale with the following criteria: “1” does not meet, “2” somewhat meets, “3” meets, “4 “is somewhat compliant, “3″ is compliant, “5″ is very compliant, and the scoring criterion: the sum of 31 items. The higher the score, the higher the level of PBT. This scale is designed for the Chinese population and has not been applied to other populations. In this study, the Cronbach’s alpha coefficient of this table was 0.875.

#### Postpartum post-traumatic stress disorder

This scale was sinicized and applied by Yang Xiaoyun (18). The scale includes four dimensions: trauma re-experiencing symptoms, avoidance numbing symptoms, and increased alertness symptoms, with a total of 17 items. It has been widely used in clinical studies investigating maternal PP-PTSD. According to the severity of symptoms, Likert’s 5-point (1–5 points) scale was used, with a total score of 17–85 points, the higher the score, the higher the likelihood of PP-PTSD, and the total score of ≥38 points was considered to be positive for PP-PTSD.

The content of the questionnaires was designed in an electronic form using the “Sojump” website.^1^ Participants were provided with QR codes for direct scanning, facilitating completion through WeChat. Upon submission, questionnaires were automatically transmitted to the Wenjuanxing platform. The questionnaires were distributed by the researchers themselves, using a standardized procedure to obtain informed consent. A total of 310 questionnaires were collected, but 4 questionnaires with a completion time of less than 100 s were excluded, resulting in 306 valid questionnaires and a response rate of 98%. The specific identity information of the research object does not appear in the study, and the data is only used for this study. In order to control confounding factors, two people checked the data quality in the process, and screened out the problems such as outliers and missing values in the original data to improve the prediction effect of the model.

### Statistical analysis

Data analysis was performed using SPSS 23.0 statistical software (IBM, United States). Normally distributed continuous variables are presented as mean ± standard deviation (SD) and were compared using independent sample *t*-tests and analysis of variance (ANOVA) for between-group comparisons. Categorical variables were presented as counts and percentages. Pearson correlation analysis explores the correlation between PBT and PP-PTSD. Perform multiple linear regression analysis on items with a *p*-value <0.05 in the uni-variate analysis, The significance level was set at *α* = 0.05.

## Results

The characteristics and demographics of the participants are shown in Table 1. A total of 306 on women who had a vaginal delivery were enrolled, with an average age of 30.14 ± 3.5 years. The average score of PBT at 3 and 42 days postpartum were (43.37 ± 9.46) and (51.40 ± 13.54) respectively, the difference was statistically significant (*p* < 0.05).

**Table 1:** Characteristics of the study population.

Item	Group	*N* (%)
Age	<30 years	144 (47)
	≥30 years	162 (53)
Education	College degree and below	144 (47)
	Bachelor degree and above	162 (53)
Work situation	Individual merchants	9 (2)
	Freelance	225 (74)
	Unemployed	17 (6)
	Public institutions or officials	55 (18)

There were statistically significant differences in the dimensions of medical support trauma perception, labor pain trauma perception, family support trauma perception and delivery outcome trauma perception (*p* < 0.05). The average score of PP-PTSD at 3 and 42 days postpartum were (22.38 ± 7.13) and (22.29 ± 5.77) respectively, with no statistical significance (*p* > 0.05).

The positive rate of PP-PTSD (score ≥ 38) at 3 and 42 days postpartum were 5 and 2%, respectively (Table 2).

**Table 2:** Score of PBT, the 4 dimensions and PP-PTSD at 3 and 42 days postpartum.

Dimension	3 days postpartum	42 days postpartum	Test statistic	*p*-value
BTPS-WVD	43.37 ± 9.46	51.40 ± 13.54	−8.508	<0.001
PP-PTSD	22.38 ± 7.13	22.29 ± 5.77	0.168	0.866
Medical support trauma	15.77 ± 3.76	18.88 ± 6.21	−7.484	<0.001
Delivery pain trauma	12.98 ± 4.39	15.81 ± 5.80	−6.798	<0.001
Family support trauma	6.45 ± 1.38	7.27 ± 1.99	−5.893	<0.001
Delivery outcome trauma	8.17 ± 3.48	9.45 ± 3.91	−4.29	<0.001

The results showed that there existed higher scores of PBT at 42 days postpartum, and in order to understand the influencing factors of PBT at 42 days postpartum, univariate analysis was conducted to analyze the influencing factors, and the results showed that, feeding mode, the effect of breast swelling on mood, mother separate from the newborn, separation time between mother and newborn, place of puerperium, psychological discomfort caused by delivering with others, use of epidural anesthesia, delivery time, advise others to deliver vaginally, the effects of wound pain, time of the postnatal wound pain and who decides on abnormal delivery were independently associated with PBT (*p* < 0.05) (Table 3).

**Table 3:** Characteristics of the study population and univariate analysis of PBT at 42 days postpartum.

Variables	Classifications	*N* (%)	Score of BTPS-WVD (mean ± SD)	Test statistic	*p*-value
Age	<30 years	144 (47)	52.90 ± 13.47	*t* = 1.835	0.067
	≥30 years	162 (53)	50.07 ± 13.51		
Education	College degree and below	144 (47)	50.32 ± 13.11	*t* = −1.320	0.188
	Bachelor degree and above	162 (53)	52.36 ± 13.89		
Work situation	Individual merchants	9 (2)	44.44 ± 12.89	*F* = 2.418	0.066
	Freelance	225 (74)	54.67 ± 12.50		
	Unemployed	17 (6)	54.47 ± 11.71		
	Public institutions or officials	55 (18)	50.65 ± 13.80		
Payment’s methods	Resident medical insurance	57 (19)	54.04 ± 12.60	*F* = 1.415	0.245
	Maternity insurance	233 (76)	50.89 ± 13.88		
	Out-of-pocket	16 (5)	49.44 ± 10.97		
Work during pregnancy	Yes	217 (71)	50.78 ± 13.88	*t* = −1.249	0.213
	No	89 (29)	52.91 ± 12.62		
Exercise during pregnancy	Yes	185 (60)	51.61 ± 13.40	*t* = 0.325	0.746
	No	121 (40)	51.09 ± 13.80		
Understanding about childbirth	Almost unknown	24 (8)	51.71 ± 13.99	*F* = 0.437	0.646
	Partial	196 (64)	51.87 ± 13.29		
	Familiar	86 (28)	50.24 ± 14.06		
Feeding mode	Breast feeding	133 (44)	48.75 ± 11.55	*F* = 5.690	0.004
	Formula feeding	154 (50)	53.95 ± 14.97		
	Mixed feeding	19 (6)	42.96 ± 10.56		
Feeding methods	Breast	85 (28)	48.84 ± 11.18	*F* = 2.698	0.069
	Bottle + Breast	202 (66)	52.68 ± 14.53		
	Bottle	19 (6)	49.26 ± 10.56		
Estimated breastfeeding time	0	19 (6)	49.26 ± 10.56	*F* = 0.279	0.757
	0–1 year	177 (58)	51.40 ± 13.13.85		
	≥1 year	110 (36)	51.78 ± 13.57		
Breast swelling	Yes	251 (82)	51.32 ± 13.48	*t* = −0.218	0.827
	No	55 (18)	51.76 ± 13.94		
The effect of breast swelling on mood	Almost none	114 (37)	49.01 ± 13.48	*F* = 4.204	0.016
	Partial	146 (48)	51.95 ± 13.99		
	Significant	46 (15)	55.61 ± 11.02		
Mother separate from the newborn	Yes	53 (17)	55.23 ± 15.37	*t* = 2.042	0.045
	No	253 (83)	50.60 ± 13.02		
Separation time between mother and newborn	not have	250 (82)	50.69 ± 13.04	*F* = 4.562	0.011
	<7 days	26 (8)	50.19 ± 13.01		
	≥7 days	30 (1)	58.40 ± 16.32		
Place of puerperium	Maternity center	60 (19)	56.02 ± 17.01	*t* = 2.461	0.016
	Home	246 (80)	50.28 ± 12.33		
Postpartum primary caregivers	Husband	223 (73)	51.58 ± 14.41	*F* = 0.092	0.912
	Parents	49 (16)	50.67 ± 9.25		
	Maternity matron	34 (11)	51.26 ± 13.11		
Delivering with others	Yes	233 (76)	51.45 ± 13.04	*t* = 0.104	0.918
	No	73 (24)	51.25 ± 15.13		
Psychological discomfort caused by delivering with others	Yes	28 (9)	59.29 ± 12.11	*t* = 3.284	0.001
	No	278 (91)	50.61 ± 13.44		
Use of epidural anesthesia	Yes	193 (63)	52.80 ± 14.03	*t* = 2.464	0.014
	No	113 (37)	49.01 ± 12.36		
Use of Doula accompaniment	Yes	204 (67)	52.18 ± 13.78	*t* = 1.462	0.155
	No	102 (33)	49.84 ± 12.97		
Medical intervention	Yes	71 (23)	50.28 ± 13.59	*t* = −0.795	0.427
	No	235 (77)	51.74 ± 13.54		
Abnormal fetal heart rate	Yes	162 (53)	50.81 ± 13.04	*t* = −0.804	0.422
	No	144 (47)	52.06 ± 14.10		
Number of vaginal examinations	1–3 times	157 (51)	50.16 ± 12.59	*F* = 1.368	0.256
	4–6 times	120 (39)	52.78 ± 14.44		
	7 or more	29 (10)	52.45 ± 14.49		
Delivery time	1–5 h	159 (52)	49.06 ± 12.69	*F* = 5.140	0.006
	6–10 h	90 (29)	54.19 ± 14.14		
	11 h and over	57 (19)	53.54 ± 13.92		
Willingness to share delivery experiences	Yes	299 (98)	51.43 ± 13.50	*t* = 0.249	0.804
	No	7 (2)	50.14 ± 16.23		
Advise others to deliver vaginally	Yes	235 (77)	50.28 ± 13.47	*t* = −2.661	0.008
	No	71 (23)	55.11 ± 13.19		
The effects of wound pain	Light	166 (54)	48.59 ± 12.76	*F* = 19.60	<0.001
	Partial	108 (35)	52.00 ± 13.20		
	Seriously	32 (11)	63.97 ± 11.37		
Time of wound pain	1–2 days	65 (21)	44.20 ± 9.88	*F* = 12.562	<0.001
	3–4 days	107 (35)	53.42 ± 15.33		
	5 days and more	134 (44)	53.28 ± 12.41		
Follow up the wound at the hospital	Yes	38 (12)	49.71 ± 9.73	*t* = −1.076	0.286
	No	268 (88)	51.64 ± 13.99		
Be respected by the hospital	Yes	70 (23)	52.63 ± 13.29	*t* = 0.863	0.389
	No	236 (77)	51.04 ± 13.62		
Who decides on abnormal delivery	Oneself	273 (89)	50.767 ± 13.50	*t* = −2.918	0.006
	Family	33 (11)	57.45 ± 12.51		

A multiple linear regression analysis was conducted on the influencing factors of PBT in women, with the total score of PBT as the dependent variable and statistically significant indicators in uni-variate analysis as independent variables. The specific assigned values are shown in Table 4. The factors that were ultimately included in the regression equation include mother separate from the newborn, separation time between mother and newborn, place of puerperium, psychological discomfort caused by delivering with others, the effects of wound pain, time of wound pain, who decides on abnormal delivery. The adjustment determination coefficient R2 of the fitting model is 0.297, indicating that the fitting model can analyze 29.7% of the variability. The overall test of the fitting model is *p* < 0.05, indicating that the fitting effect of the model is good, as shown in Table 5.

**Table 4:** Variable assignment.

Variable	Assigned method
PBT score	Original numerical values
Mother separate from the newborn	Yes = 1, No = 2
Separation time between mother and newborn	0 = dummy variable, 0–7 days (1, 0), ≥7 days (0, 1)
Psychological discomfort caused by delivering with others	Yes = 1, No = 2
Use of epidural anesthesia	Yes = 1, No = 2
Advise others to deliver vaginally	Yes = 1, No = 2
Who decides on abnormal delivery	Self = 1, Family = 2
Place of puerperium	Maternity center = 1, home = 2
Time of wound pain	1–2 days = dummy variable, 3–4 days (1,0), 5 days and more (0,1)
The effects of wound pain	Light = dummy variable, partial (1,0), seriously (0,1)
Delivery time	1–5 hours = dummy variable, 6–10 h (1,0), 11 h and above (0,1)
Feeding mode	Breast feeding = dummy variable, formula feeding (1, 0), mixed feeding (0, 1)
The effect of Breast swelling on mood	Almost none = dummy variable, partial (1, 0), significant (0, 1)

**Table 5:** Univariate analysis of PBT at 42 days postpartum.

Independent variable	Regression coefficient	Standardized effect	Test statistic	*p*-value	95% CI lower limit	95% CI upper limit
(Constant)	90.865		5.363	0	57.517	124.213
Mother separate from the newborn	−17.073	−0.478	−2.195	0.029	−32.385	−1.761
Psychological discomfort caused by delivering with others	−5.247	−0.112	−2.115	0.035	−10.13	−0.363
Use of epidural anesthesia	−1.633	−0.058	−0.992	0.322	−4.875	1.608
Advise others to deliver vaginally	−0.211	−0.007	−0.117	0.907	−3.765	3.342
Who decides on abnormal delivery	8.178	0.188	3.138	0.002	3.048	13.308
Puerperal Places	−6.102	−0.179	−3.057	0.002	−10.031	−2.173
Time of wound pain (reference: 1–2 days)						
3–4 days	6.872	0.242	3.589	<0.001	3.103	10.64
5 days and more	4.052	0.149	2.011	0.045	0.087	8.017
The effects of wound pain (reference: Light)						
Light	3.311	0.117	2.022	0.044	0.087	6.535
Seriously	14.464	0.327	5.142	<0.001	8.927	20
Delivery time (reference: 1–5 hours)						
6–10 h	0.974	0.033	0.551	0.582	−2.507	4.456
11 h and over	1.619	0.047	0.797	0.426	−2.38	5.618
Feeding mode (reference: breast feeding)						
Formula feeding	0.396	0.007	0.125	0.901	−5.855	6.647
Mixed feeding	2.508	0.093	1.571	0.117	−0.635	5.651
The effect of breast swelling on mood (reference: almost none)						
Partial	−0.851	−0.031	−0.501	0.616	−4.192	2.49
Significant	1.028	0.027	0.426	0.67	−3.718	5.775
Separation time between mother and newborn (reference: 0)						
0–7 days	−14.562	−0.3	−2.01	0.045	−28.818	−0.305
≥ 7 days	−10.825	−0.238	−1.327	0.186	−26.883	5.234

**Figure 1:**
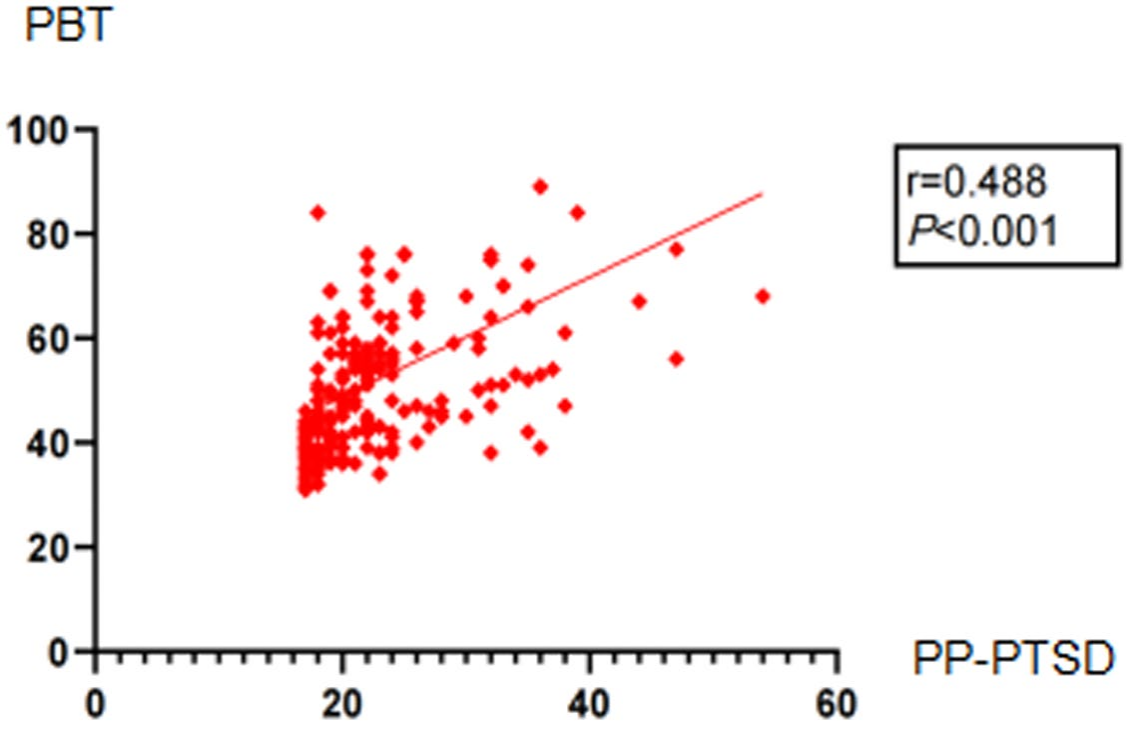
Pearson correlation analysis.

Pearson correlation analysis showed that, PBT and its four dimensions were positively correlated with PP-PTSD (*r* = 0.488, *p* < 0.001) (Figure 1; Table 6).

**Table 6:** Correlation analysis between four dimensions and PP-PTSD (*r*-value).

Item	Trauma on medical support	Trauma on labor pain	Trauma on family support	Trauma on delivery outcome
PP-PTSD	0.296**	0.385**	0.463**	0.412**

** *p* < 0.01.

## Discussion

### Current status of PBT

Psychological birth trauma and childbirth-related posttraumatic stress disorder represent a substantial burden of disease with 6.6 million mothers and 1.7 million fathers or co-parents affected by childbirth-related posttraumatic stress disorder worldwide each year (19). The main risk factors and causes of PBT are as follows, During pregnancy, risk factors most strongly associated were depression, fear of childbirth, poor health or complications of pregnancy, history of trauma, or previous psychological therapy for pregnancy or birth-related problems. During birth, risk factors most strongly associated were negative subjective birth experiences, operative birth (assisted vaginal delivery or cesarean delivery), and dissociation (including depersonalization, derealization, and emotional numbness) (20). Considering the harm of PBT, this study further investigated the risk factors of PBT in Chinese population.

This study examined the incidence of postpartum trauma at 3 and 42 days postpartum using the BTPS-WVD scale. The findings indicated that the severity of PBT increased over time following childbirth, suggesting the need for extended care to monitor maternal mental health during this critical period. Hospitals should implement comprehensive follow-up programs, families should maintain continuous support for maternal well-being, and society should increase its involvement in providing resources and assistance. Additionally, integrating psychologists into home-based support systems can help mothers navigate this challenging period characterized by elevated PBT levels. The median PBT scores in this study were 41 and 44 at 3 and 42 days postpartum respectively, and the number of people with PBT above the median at 3 and 42 days postpartum were 55 and 61%, respectively. Due to the lack of objective diagnostic criteria at present, the incidence of PBT varies substantially among studies (21), ranging from 9 to 44%. The differences in outcomes could be attributed to the following subjective and objective factors, medical intervention, maternal and newborn safety/separation, environment, delivery expectation, loss of control, doctor-patient communication and support (22). Murphy et al. (23) interviewed 4 women with PBT experience after their first delivery through narrative research method, and the results showed that early family planning with medical staff was a protective factor to avoid reoccurrence of trauma. Greenfield et al. (24) investigated 9 pregnant women who had undergone PBT and had a second pregnancy using longitudinal grounding theory and discovered that they had a strong desire to avoid suffering PBT again. They agreed that having access to credible information from specialists and collaborating with professionals to create and confirm pregnancy and family planning in the early stages of pregnancy were helpful approaches to avoid additional trauma.

The average score of PP-PTSD at 3 and 42 days postpartum was no statistical significance, with positive rates of 5 and 2%, respectively. In the UK and Australia, the prevalence of PP-PTSD in large-sample studies ranges from 1 to 6% (25, 26), Wang Meifang (27) conducted PP-PTSD surveys at 1–3, 4–6, and 7–12 months postpartum. The results showed that the positive rates of PP-PTSD were 10.8%, 12.6%, and 15.1%, respectively. The differences in positive PP-PTSD rates over the three periods were not statistically significant, implying that postpartum women’s PP-PTSD status remained rather consistent over the course of a year. However, the findings of this study suggest the incidence of post-traumatic stress disorder steadily falls after delivery, indicating that the severity of post-traumatic stress disorder decreases over time. Possible reasons include the body’s recuperation following childbirth, which enables it to adequately care for both itself and the newborn. As a result, it is critical to aid postpartum women in recuperating physically and mentally throughout the postpartum period in order to reduce the detrimental influence of the traumatic event during childbirth on their psychological health. This study found that the four components of the BTPS-WVD Scale, namely medical support, labor pain, family support, and delivery outcome, are all positively connected with post-traumatic stress disorder (PTSD). The ERTAN (8) study also indicated that birth trauma is a risk factor for postpartum traumatic stress disorder, which is in line with the findings of this study. As a result, all four dimensions of birth trauma deserve account, and interventions before and after labor can be based on these four components.

### Analysis of the influencing factors of PBT at 42 days postpartum

#### Parturition factors

This study found that risk factors for PBT include delivering with others and who decides the abnormal delivery mode. The higher the PBT score, the more significant the psychological impact on parturients. In addition, women who experienced higher levels of trauma during vaginal delivery were less likely to recommend the same delivery to others. Dai Ling (28) conducted a study in which pregnant women observed other pregnant women’s poor birthing experiences and emergency. They experience emotional breakdowns and lose psychological resilience as a result of the tight atmosphere and fear of encountering the same situation. Beck (29) and Meyer (30) et al. argue that the loss of dignity is a key aspect in delivery trauma, and disrespect during labor can have an influence on a woman’s mental health. According to a study, during delivery, pregnant women have low levels of awareness and lack decision-making power, which can make them feel helpless. The study also found that the sense of losing control in decision-making and the lack of sufficient information are predictors of childbirth trauma (31). A Spanish study (32) revealed that unexpected birth, severe pain, lack of informed consent, and lack of social support are factors that give rise to PBT. Iranian researchers Taghizadeh et al. (33) carried out semi-structured interviews with 23 women ranging from 3 days postpartum to 32 years postpartum and discovered that the absence of delivery education, unfamiliar delivery environment, mother and child threatened, poor doctor-patient communication, and insufficient family support were significant risk factors for PBT. In the review carried out by Watson et al. (34), all interviewees disclosed having PBT experience. The study discovered that inappropriate care delivered by healthcare personnel, such as medical staff negligence, excessive medical intervention, etc., the loss of control and decision-making power during delivery, and insufficient social support were associated with PBT. Therefore, it is recommended that clinical medical staff pay heed to the PBT’s parturition factors, enhance delivery management, respect the demands of pregnant women, improve the medical experience, and promote the physical and mental health of pregnant women.

#### Postpartum factors

Among postpartum factors, formula feeding, separation time from newborn for at least 7 days, time of wound pain for at least 5 days, place of puerperium was Maternity center, the effect of Breast swelling on mood was significant had higher PBT scores. Chan (35) et al. also pointed out that the mother’s PBT is associated with the baby’s attachment, and being separated from the baby after delivery is a cold and dreadful experience that can make her feel lonely and sorrowful. Li Tao’s (2) research also referred that the lack of maternal–infant relationships after delivery and the transfer of the newborn to the NICU can make the mother feel lonely and ignored, thereby affecting her mental health.

Several studies (32, 36) have demonstrated that breastfeeding serves as a protective factor against PBT, which had the same findings as in this study. As breastfeeding may stimulate the secretion of hormones in women in vaginal delivery, it leads to more positive emotions and less stress (37). It is crucial to offer breastfeeding guidance during the prenatal, intrapartum and postpartum phases for women who have undergone maternal–infant separation.

Maternity centers are staffed by nutritionists, obstetricians, pediatricians, and postpartum therapists, so women in vaginal delivery and their families generally believe that maternity centers are more conducive to maternal recovery than at home. However, this study actually discovered that the postpartum period spent in a maternity center was ineffective in helping women reduce their PBT. This could reverse women’s perception of the postpartum care method. The absence of family support constitutes a detrimental factor that influences the mental health of women in vaginal delivery (38). A lack of supportive, listening and supportive communication from a partner can increase a woman’s negative experience of delivery (39). It is more beneficial to reduce the PBT for women in vaginal delivery by encouraging family members to offer greater support and encouragement to them and creating a warm and supportive family environment after delivery. This study reveals that the 42 days postpartum is associated with a higher PBT. Hence, healthcare workers should pay greater attention to this stage and collaborate with families and society to mitigate the impact of risk factors and facilitate women in vaginal delivery faster physical and mental recovery.

### Intervention measure for PBT

The research on PBT can be traced back to as early as 1996, according to a study (5). Furthermore, there has been a consistent increase in the number of papers published in this field over the past 3 years, indicating an enduring scholarly interest in maternal psychology. The impact of PBT extends beyond the physical and mental health of the women, encompassing her family, marital relationship, and even society itself. Therefore, it is imperative to address this global public health issue (22). The identification of the factors that contribute to PBT during delivery can enable the early identification of high-risk groups, the formulation of intervention strategies, the improvement of maternal mental health management, and the advancement of the health of both mothers and infants. The interventions referred to in the previous literature are as follows: Enlarging prenatal education to enhance maternal knowledge and improve psychological resilience, thereby lowering the incidence of PBT during delivery (30). Strengthening maternal family support and medical assistance, providing spouses with intimate physical contact and continuous support (40), can contribute to mitigating the degree of PBT. Previous meta-analyses demonstrate that proactive and early psychological intervention implemented by healthcare providers within 72 h postpartum proves effective in alleviating PBT symptoms in women during the 4–6 weeks postpartum period (41). Iranian scholar Hajarian Abhari et al. (42) offered Gamble psychological counseling at 35, 36, 37 weeks, and 4–12 h postpartum. Each session centered on: establishing a therapeutic relationship with women in vaginal delivery, introducing prenatal and postpartum problems, supporting the expression of emotions, facilitating the establishment of a connection between behavior, emotions, and delivery, and encouraging the sharing of the delivery. The results demonstrated that Gamble psychological counseling could effectively reduce the incidence of PBT after delivery. Taheri et al. (43) undertook semi-structured interviews with pregnant women, spouses, and healthcare professionals to summarize the prevention strategies for PBT, which comprise, intensifying prenatal training, providing a conducive delivery environment, appropriate care measures, such as pain alleviation and avoidance of excessive manipulation, and creating a favorable working atmosphere for midwives to elevate the quality of midwifery care. At present, the intervention measures remain in the development stage. Larossa et al. (44) explored the neurobiology and attachment implications of early tactile experience and discussed the importance of early postpartum experience in mitigating birth-related trauma. According to the results of the previous study, the research team conducted a continuous study on the intervention of labor trauma in women with vaginal delivery, and the specific intervention method was to improve the PBT level through the postpartum Internet-based care integrated with extended support services. In future studies, we propose extending the follow-up periods for assessing PBT status to 3 months, 6 months, and 1 year postpartum. This will provide a more comprehensive understanding of the changes in postpartum PBT among women who have undergone vaginal delivery. This will provide theoretical underpinnings for the prevention of postpartum PBT.

In conclusion, women who experienced PBT during vaginal delivery reported significantly higher levels of perceived trauma at 42 days postpartum compared to 3 days postpartum. Mother separate from the newborn, separation time between mother and newborn, place of puerperium, psychological discomfort caused by delivering with others, the effects of wound pain, time of wound pain, who decides on abnormal delivery were its risk factors. Healthcare workers, families, and society are obligated to implement targeted and individualized nursing strategies for high-risk groups so as to diminish the perceived extent of PBT among women who had a vaginal delivery and ensure the mental well-being of women during the perinatal period. However, the study exhibited several limitations that warrant consideration. The sample was derived from a single center, and the use of convenience sampling may introduce biases, including but not limited to geographical and age distribution biases. The study did not conduct a comparative analysis between populations with high and low PBT levels. The number of factors considered in the analysis was limited, which could be expanded in future research to address these limitations more comprehensively. Lastly, The BTPS-WVD scale has only been validated in the Chinese population, suggesting the need for further research on global applicability.

## Data Availability

The raw data supporting the conclusions of this article will be made available by the authors, without undue reservation.

## Footnotes

^1^ https://www.Wjx.cn/
